# C-reactive protein and procalcitonin for antimicrobial stewardship in COVID-19

**DOI:** 10.1007/s15010-021-01615-8

**Published:** 2021-05-22

**Authors:** Isabell Pink, David Raupach, Jan Fuge, Ralf-Peter Vonberg, Marius M. Hoeper, Tobias Welte, Jessica Rademacher

**Affiliations:** 1 Department of Respiratory Medicine, Hannover Medical School, Carl-Neuberg-Str. 1, 30625 Hannover, Germany; 2 Department of Respiratory Medicine, Hannover Medical School, Member of the German Center for Lung Research (BREATH), Carl-Neuberg-Str. 1, 30625 Hannover, Germany; 3 Institute for Medical Microbiology and Hospital Epidemiology, Hannover Medical School, Carl-Neuberg-Str. 1, 30625 Hannover, Germany

**Keywords:** COVID-19, C-reactive protein, Procalcitonin, Antimicrobial stewardship, Secondary bacterial infections

## Abstract

**Purpose:**

Coronavirus disease 2019 (COVID-19) caused by severe acute respiratory coronavirus 2 (SARS-CoV-2) has spread around the world. Differentiation between pure viral COVID-19 pneumonia and secondary infection can be challenging. In patients with elevated C-reactive protein (CRP) on admission physicians often decide to prescribe antibiotic therapy. However, overuse of anti-infective therapy in the pandemic should be avoided to prevent increasing antimicrobial resistance. Procalcitonin (PCT) and CRP have proven useful in other lower respiratory tract infections and might help to differentiate between pure viral or secondary infection.

**Methods:**

We performed a retrospective study of patients admitted with COVID-19 between 6th March and 30th October 2020. Patient background, clinical course, laboratory findings with focus on PCT and CRP levels and microbiology results were evaluated. Patients with and without secondary bacterial infection in relation to PCT and CRP were compared. Using receiver operating characteristic (ROC) analysis, the best discriminating cut-off value of PCT and CRP with the corresponding sensitivity and specificity was calculated.

**Results:**

Out of 99 inpatients (52 ICU, 47 Non-ICU) with COVID-19, 32 (32%) presented with secondary bacterial infection during hospitalization. Patients with secondary bacterial infection had higher PCT (0.4 versus 0.1 ng/mL; *p* = 0.016) and CRP (131 versus 73 mg/L; *p* = 0.001) levels at admission and during the hospital stay (2.9 versus 0.1 ng/mL; *p* < 0.001 resp. 293 versus 94 mg/L; *p* < 0.001). The majority of patients on general ward had no secondary bacterial infection (93%). More than half of patients admitted to the ICU developed secondary bacterial infection (56%). ROC analysis of highest PCT resp. CRP and secondary infection yielded AUCs of 0.88 (*p* < 0.001) resp. 0.86 (*p* < 0.001) for the entire cohort. With a PCT cut-off value at 0.55 ng/mL, the sensitivity was 91% with a specificity of 81%; a CRP cut-off value at 172 mg/L yielded a sensitivity of 81% with a specificity of 76%.

**Conclusion:**

PCT and CRP measurement on admission and during the course of the disease in patients with COVID-19 may be helpful in identifying secondary bacterial infections and guiding the use of antibiotic therapy.

## Introduction

The novel beta-coronavirus SARS-CoV-2 caused a major outbreak of respiratory illness starting in Wuhan, China at the end of 2019. In February 2020, World Health Organization (WHO) named the novel coronavirus disease as COVID-19 [1]. Until 15th December 2020, more than 76 million cases and over 1.6 million deaths due to COVID-19 have been confirmed worldwide. Although the vast majority of people suffered from mild or uncomplicated illness, severe disease requiring hospitalization is also seen in a subset of patients [2].

The knowledge about symptoms, clinical course, and risk factors of COVID-19 disease is increasing [3–5], but data on co-infections and the use of antibiotic therapy are limited. Most studies did not address secondary infections [6]. In patients with mild COVID-19, bacterial co-infections are rare, but in severe disease, co-infections have been reported in up to 50% of the affected patients [6–9]. The prevalence of confirmed co-infections in patients with COVID-19 on general ward was 3.5% in a series from the US (*n* = 1705) [10], 4% in a systematic review and meta-analysis from the UK (3834 patients) [7] and 8% from another review from the UK (*n* = 806) [6]. Rates of co-infections in ICU patients varied from 14 to 50% [7, 9]. Despite the overall low rates of confirmed secondary bacterial infections, the vast majority (57–86%) of COVID-19 patients received empirical antibiotic therapy [6, 8, 10].

Serum procalcitonin (PCT) may be useful in identifying secondary infections in patients with COVID-19. In isolated COVID-19, as in other viral infections, PCT levels usually remain normal. The lack of a PCT rise in viral infections may be due to virus-stimulated production of interferon-γ by macrophages, which inhibits TNF-α in the immune response [11]. PCT has emerged as a promising tool to facilitate decisions about antibiotic therapy in lower respiratory tract infections [12–14].

An association of PCT levels with bacterial co-infections in hospitalized patients with COVID-19 has been demonstrated in a few studies [3]. Van Berkel et al. found that a PCT level of < 0.25 µg/L had a negative predictive value of 81% and a PCT > 1 µg/L had a positive predictive value of 93% for bacterial co-infection [9]. The authors concluded that antibiotic therapy can be safely withheld in patients with COVID-19 and low PCT levels on the ICU.

CRP is usually increased on presentation in patients with COVID-19 [9]. As CRP is consistently elevated, this biomarker might not have predictive value for bacterial infections in the initial phase of COVID-19. Serial CRP with an in- or decrease over time may help to identify or rule out nosocomial bacterial infections and prompt appropriate use of antibiotic therapy [9]. Patients with severe disease had also higher CRP levels than those with non-severe disease [2].

Our institution implemented a COVID-19 standard operation procedure (SOP) in March 2020 for diagnostic measures and antimicrobial treatment. The collection of two sets of blood cultures and urinary antigen test for *Legionella pneumophila* and *Streptococcus pneumoniae* in each patient with confirmed COVID-19 disease was part of the routine diagnostic workup. In case of suspicion of secondary bacterial or fungal infection a bronchoscopy with broncho alveolar lavage (BAL) or tracheal secret and urine sampling were supplemented. In non-ICU patients with PCT levels < 0.5 ng/mL, antibiotic therapy was not recommended. For ICU patients no standard recommendations regarding antibiotic therapy were made.

In the present study, we investigated the diagnostic value of PCT and CRP to detect secondary bacterial infections in patients with COVID-19.

## Materials and methods

### Study design and participants

**Fig. 1:**
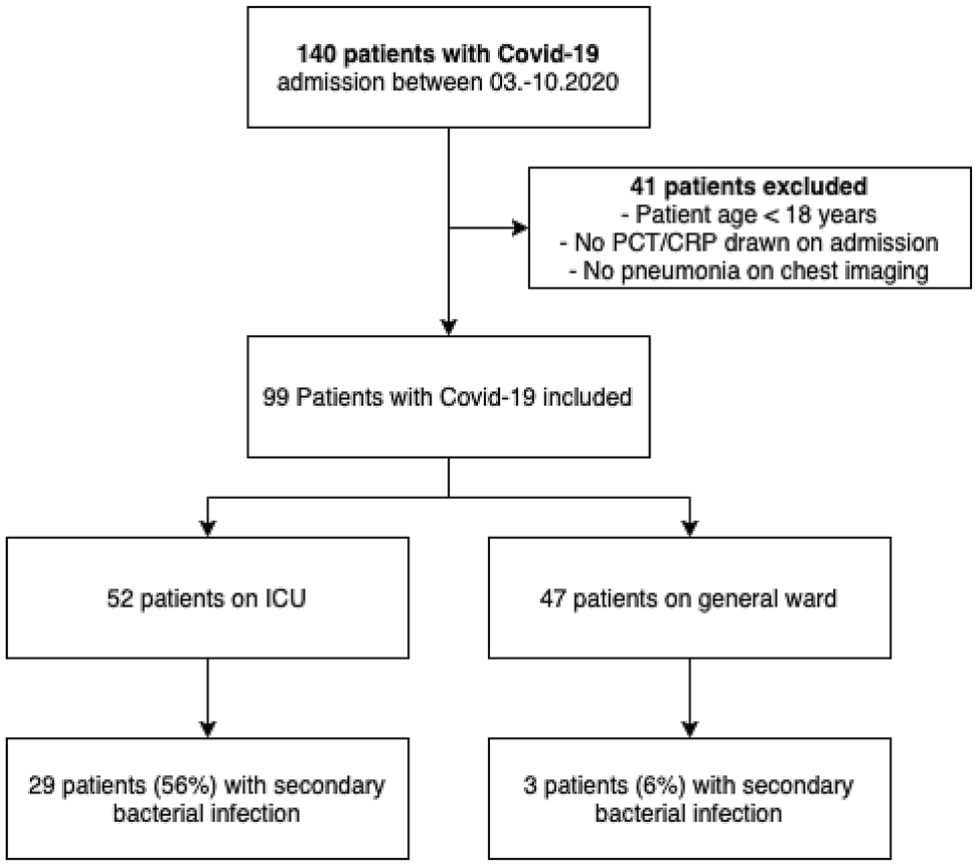
Flow diagram of study cohort

This was a retrospective single centre study which has been approved by the internal review board (No. 9491_BO_K_2020). All hospitalized patients aged 18 years and older with confirmed Covid-19 pneumonia, admitted between 6th March and 30th October 2020 to a large university hospital, located in Hannover (Hannover Medical School, MHH), Germany, were included in the study (Fig. 1). The final date of follow up was 15th December 2020. Patient characteristics including baseline demographics and chronic comorbidities were obtained. PCT and CRP levels were obtained on admission and on every second day on general ward and daily on the ICU. Data were recorded according to good clinical practice (GCP) guidelines including patient demographics, comorbidities, clinical parameters, laboratory findings, microbiology, outcome, antibiotic, antifungal and antiviral regime. Laboratory confirmation of SARS-CoV-2 was achieved by reverse transcription polymerase chain reaction (RT-PCR). All patients underwent radiological chest examination to confirm pneumonia. Patients without PCT or CRP results were excluded from the study as patients without evidence for pneumonia on their chest radiograph. Urine samples were used for lateral flow antigen test detection of *L. pneumophila* serogroup 1 and *S. pneumoniae* (AlereBinaxNOW, Abbott, Wiesbaden, Germany). For microbiological workup, blood was inoculated into aerobic and anaerobic Blood culture (BC) media via an automated BC system (Bactec, Becton Dickinson GmbH, Heidelberg, Germany). Culture bottles were incubated for 5–7 days according to the manufacturer’s recommendations. Considering the difficulty in determining the clinical significance of *coagulase-negative staphylococci* (CoNS) in BC, these isolates were reviewed separately based on the number of positive culture sets, the presence of intravascular devices, and patient’s characteristics. Isolates were considered clinically significant (true bacteremia) if two or more bottles yielded the same CoNS. Respiratory samples (tracheal secret or BAL) for microbiological tests were obtained and cultures were performed on Columbia sheep blood agar, chocolate agar, and sabouraud dextrose agar (Becton Dickinson GmbH, Heidelberg, Germany). In the case of a clinically suspected infection by Legionella, a buffered charcoal yeast extract (BCYE) agar was used in addition (Becton Dickinson GmbH, Heidelberg, Germany).

The primary outcome of this study was secondary bacterial infections (yes or no) and associated PCT and CRP levels. Secondary bacterial infection was defined as “any infectious episode” evidenced by the presence of a bacterial pathogen in positive blood cultures, respiratory samples or urine. A specialist in infective medicine not involved in the patient’s care classified the clinical relevance of the detected pathogen. In case of multiple secondary bacterial infections, only the first infectious episode was analysed. The patients were divided into two groups according to the presence or absence of secondary bacterial infection. Disease severity was classified based on the place of treatment (ICU/Non-ICU) and the WHO Ordinal Scale for Clinical Improvement [15].

### Statistical analysis

IBM SPSS Statistics (version 27.0, IBM Corp., Armonk, New York) and Stata 13.0 (State Corp LP, College Station, Texas, USA) statistical software programs were used for statistical analysis. Categorical data were presented as numbers and percentages, and for group comparisons, Chi-square, or Fisher’s exact test were used as appropriate. Continuous variables were presented as medians with the first and third quartile (Q1 and Q3), and for group comparisons two-sided *t* test or Mann–Whitney *U* test were used as appropriate. Receiver operating characteristic (ROC) analysis was performed to assess the predictive performance of PCT resp. CRP and secondary bacterial infection and to calculate the AUC. Using ROC curve analysis with calculation of the area under curve (AUC), the best cut-off value of PCT and CRP with the corresponding sensitivity and specificity were calculated. All tests were two-sided; a *p* value of ≤ 0.05 was considered statistically significant [16].

## Results

### Demographic, clinical information and laboratory findings

During the study period, 140 confirmed COVID-19 patients were evaluated for the study. After exclusion of patients < 18 years, no PCT/CRP on admission or no pneumonic infiltrates on chest imaging, 99 patients were eligible for this analysis (Fig. 1), of whom 52 were treated on ICU and 47 on general ward. COVID-19 was diagnosed by RT-PCR in all patients. The median age of the cohort was 57 years (range 18–91), and 72 (73%) patients were male. Comorbidities comprised of obesity (BMI ≥ 30), any history of cardiac disease, pulmonary disease, hypertension, diabetes mellitus, renal disease, liver disease, any kind of immunosuppression, solid organ transplantation, and malignancies. Arterial hypertension, diabetes, and coronary heart disease were the most frequent comorbidities. Only a minority of 16% had no comorbidities.

The median interval between symptom onset and hospital admission was 7 days. PCT and CRP levels on admission and during the hospital stay were higher in ICU patients. During hospitalization, 19 patients died. Detailed characteristics of the subjects and laboratory findings are shown in Table 1.

**Table 1:** Patients characteristics and laboratory findings of 99 patients with COVID-19 pneumonia

	Total cohort (*n* = 100)	ICU COVID-19 patients (*n* = 52)	Non-ICU COVID-19 patients (*n* = 47)
Median age (range)	57 (18–91)	55 (18–82)	58 (18–91)
Male sex	72 (73%)	45 (87%)	27 (57%)
Median WHO scale	5 (3–8)	7 (5–8)	3 (3–8)
Comorbidities			
No comorbidities as listed below	16 (16.2%)	5 (9.6%)	11 (23.4%)
Obesity	10 (10.1%)	8 (15.4%)	2 (4.3%)
Arterial Hypertension	39 (29.4%)	23 (44.2%)	16 (34%)
Diabetes	19 (19.2%)	15 (28.8%)	4 (8.5%)
Coronary heart disease	17 (17.2%)	7 (13.5%)	10 (21.3%)
Congestive heart failure	3 (3%)	2 (3.8%)	1 (2.1%)
COPD	8 (8.1%)	1 (2.1%)	8 (8.1%)
Asthma	2 (2%)	1 (1.9%)	1 (2.1%)
Chronic kidney disease	3 (3%)	1 (2.1%)	2 (3.8%)
Cancer	8 (8.1%)	5 (9.6%)	3 (6.4%)
Immunodeficiency	4 (4%)	1 (1.9%)	3 (6.4%)
Organ transplantation	5 (5.1%)	2 (3.8%)	3 (6.4%)
Chronic liver disease	4 (4%)	2 (3.8%)	2 (4.3%)
Antifungal therapy	12 (12%)	12 (23.1%)	0 (0%)
Antiviral therapy	32 (32%)	24 (46.2%)	8 (17%)
Antibiotic therapy	68 (68.7%)	49 (94%)	19 (40.4%)
Median interval between symptoms and hospital admission (days)	7 (6–9)	7.5 (7–13.3)	7 (5–7)
Secondary bacterial infection	32 (32.3%)	29 (55.8%)	3 (6.4%)
Nosocomial/community acquired infection	84.3%/ 15.7%	82.8%/ 17.2%	100%/0%
Laboratory findings			
PCT (ng/mL) on admission	0.2 (0.1–0.4)	0.35 (0.2–0.9)	0.1 (0.1–0.1)
Highest PCT (ng/mL)	0.3 (0.1–2.4)	1.75 (0.1–80)	0.1 (0.1–0.2)
Day of highest PCT after dmission	1 (1–3)	3 (1–7.5)	1 (1–2)
CRP (mg/L) on admission	84.4 (42.7–147.2)	122 (77.5–185,1)	52.4 (16.8–93.5)
Highest CRP (mg/L)	135 (73.8–220)	193.5 (133.3–332.8)	73.8 (26–126)
Day of highest CRP after admission	2 (1–5)	3 (2–9)	1 (1–3)
Deceased during hospital stay	19 (19.2%)	15 (28.8%)	4 (8.5%)

Data are presented as absolute numbers and relative frequencies (*n*(%)) or as median (IQR)

*COVID-19* coronavirus disease 2019; *ICU* intensive care unit; *WHO* World health organisation; *COPD* chronic obstructive pulmonary disease; *CRP* C-reactive protein; *PCT* procalcitonin

Most patients received antibiotic therapy within 24 h of admission (*n* = 68; 69%). More patients on ICU were treated with antibiotics than on general ward (94% versus 40%). The antibiotic regimens most commonly used in the ICU were Piperacillin/Tazobactam in 26 patients, followed by Meropenem in 22 patients and Ampicillin/Sulbactam in 14 patients. On general ward, the most common antibiotic therapy was Piperacillin/Tazobactam in eight patients, followed by Ampicillin/Sulbactam in five patients and Azithromycin in four patients. The majority of patients (*n* = 55; 81%) received more than one antibiotic regimen during their hospital stay. Thirty-two patients (32%) were treated with remdesivir as antiviral therapy.

### Microbiologic results

Most patients received a complete microbiological workup during their hospital stay according to local SOP. Blood cultures (BC) were collected from 87 patients (88%) with a positive rate of 14% (12 patients, after exclusion of presumed contaminations). The most common bacterial pathogen in BC was coagulase-negative staphylococci (*n* = 5), after excluding possible contaminations. All of these were catheter associated blood stream infections. Respiratory samples (tracheal secret or BAL) for microbiological tests were obtained in 44 patients (44%). BAL samples were obtained from 79% of the patients admitted to the ICU with a positive result in 29%. The most common pathogens in respiratory samples were *Escherichia coli *(*n* = 5)*, Klebsiella pneumoniae* (*n* = 4), *Haemophilus influenzae* (*n* = 4) and *Staphylococcus aureus* (*n* = 3). Four patients had clinically relevant pathogens in urine (*Pseudomonas aeruginosa* (2), *Klebsiella pneumoniae* (1) and *Escherichia coli* (1)). Urinary antigen tests for *L. pneumophila and S. pneumoniae* were performed for 74 patients (75%), with negative results in all patients. Table 2 summarizes the results of the microbiological testing from the study.

**Table 2:** Results of microbiologic diagnostics

	Total cohort	ICU COVID-19 patients (*n* = 52)	Non-ICU COVID-19 patients (*n* = 47)
Blood cultures collected	87 (87.9%)	51 (98.1%)	36 (76.6%)
Blood cultures positive	19 (19.2%)	17 (32.7%)	2 (4.3%)
Contamination only	3 (3%)	2 (3.8%)	1 (2.1%)
Bronchoscopy performed	44 (44.4%)	41 (78.8%)	3 (6.4%)
Positive result BAL or TS	15 (15.2%)	15 (28.8%)	0
*L. pneumophila* + *S. pneumoniae* urinary test performed; positive rate	74 (74.7%); 0	45 (86.5%); 0	29 (61.7%); 0
Clinically relevant bacterial pathogens in BAL/TS	*Haemophilus influenzae* (*n* = 4), *Pseudomonas aeruginosa* (*n* = 2), *Klebsiella pneumoniae* (*n* = 4), *Acinetobacter baumannii* (*n* = 1), *Staphylococcus aureus* (*n* = 3), *Escherichia coli* (n = 5), *Enterobacter cloacae* (*n* = 1), *Serratia marcescens *(*n* = *1*)*, Aspergillus fumigatus* (*n* = 1)		
Clinically relevant bacterial pathogens in BC	*Pseudomonas aeruginosa* (*n* = 1), *Klebsiella pneumoniae* (*n* = 1), *Escherichia coli* (*n* = 3), *Staphylococcus epidermidis* (*n* = 5), *Staph. hominis *(*n* = 1),* Enterococcus faecium* (*n* = 1), *Candida albicans* (*n* = 3), *Candida glabrata* (*n* = 1)		
Clinically relevant bacterial pathogens in urine	*Pseudomonas aeruginosa* (*n* = 2), *Klebsiella pneumoniae* (*n* = 1), *Escherichia coli* (*n* = 1)		

Data are presented as absolute numbers and relative frequencies (*n*(%))

*BAL* broncho alveolar lavage; *TS* tracheal secret; *BC* blood culture

### Analysis of secondary bacterial infections

Around one-third of patients (32%) developed secondary bacterial infection during hospitalization. Patients with secondary bacterial infection had higher PCT and CRP levels on admission and during their hospital stay. Details are shown in Table 3. The time of highest PCT and CRP levels correspond with the diagnosis of secondary bacterial infection.

**Table 3:** Procalcitonin and CRP levels of COVID-19 patients who did and did not develop a secondary bacterial infection

	Secondary infection	No secondary infection	*p* value
All patients	32 (32.3%)	67 (67.7%)	
PCT (ng/mL) on admission	0.4 (0.1–1.1)	0.1 (0.1–0.2)	0.016
PCT (ng/mL) highest value	2.9 (0.9–15.8)	0.1 (0.1–0.4)	< 0.001
Day of highest PCT	4.5 (1–10.8)	1 (1–2)	< 0.001
Rise/Fall of PCT (ng/mL) per day	0.2 (0–1.1)	0 (0–0.03)	0.011
CRP (mg/L) on admission	130.6 (68.8–186.65)	73.4 (31.2–119.5)	0.001
CRP (mg/L) highest value	292.5 (183.5–341.8)	93.9 (50–171)	< 0.001
Day of highest CRP	6 (2.3–11.8)	2 (1–3)	< 0.001
Rise/Fall of CRP (mg/L) per day	15.2 (4.1–28.6)	2.8 (0–10)	0.002
Deceased during hospital stay	11 (34.4%)	8 (11.9%)	0.008

Data are presented as absolute numbers and relative frequencies (*n*(%)) or as median (IQR)

COVID*-19* coronavirus disease 2019; *CRP* C-reactive protein; *PCT* procalcitonin

Only three patients (6.3%) with COVID-19 admitted to general wards had secondary bacterial infection. All other non-ICU patients had no secondary bacterial infections (*n* = 45; 93.8%). In 41 of these patients, PCT levels on admission were < 0.55 ng/mL while CRP was elevated (70.7 mg/L, IQR 2.4–96.2). Of note, 40% of these patients received empirical antibiotic therapy.

Half of ICU patients had secondary bacterial infection (55.8%). In all of these patients, PCT levels on highest value were > 0.55 ng/mL. Regarding ICU patients without secondary bacterial infection median PCT levels were 0.5 (IQR 0.3–2.4) while CRP was considerably elevated (155.8, IQR 78.8–204.6).

**Fig. 2:**
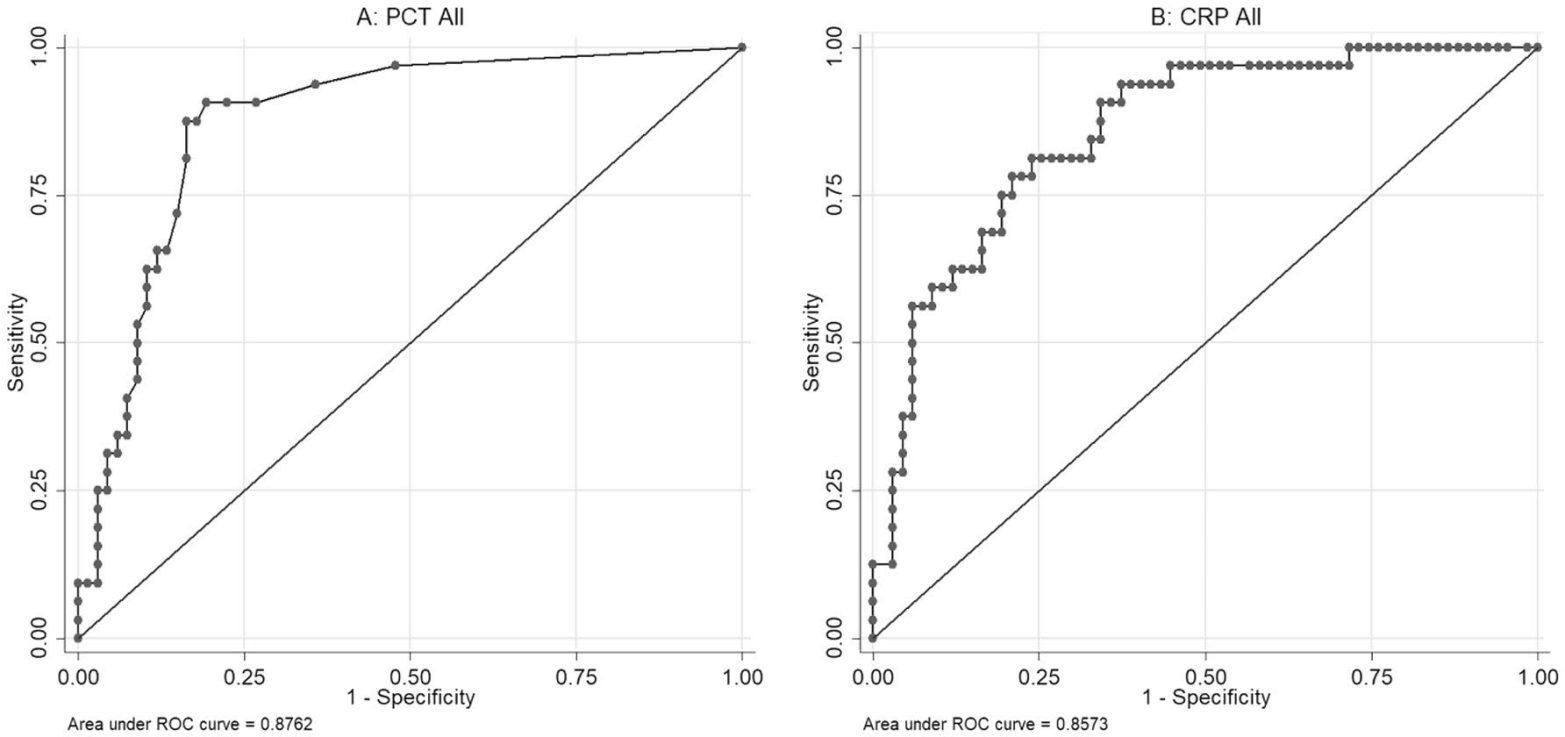
ROC curve analysis for highest PCT level (**a**) and CRP level (**b**) as a marker for secondary infection in inpatients with COVID-19 pneumonia: Analysis revealed an area under the curve of 0.88 (*p* < 0.001) for PCT and 0.86 (*p* < 0.001) for CRP

ROC analysis of PCT yielded AUCs of 0.88 (*p* < 0.001) for all patients (Fig. 2a). At a cut-off value of 0.55 ng/mL, PCT had a sensitivity of 91% and a specificity of 81% for the detection of secondary bacterial infection. In patients with PCT < 0.55 ng/mL, the negative predictive value was 94%, whereas PCT levels of > 0.55 ng/mL had a positive predictive value of 69%.

ROC analysis of CRP yielded AUCs of 0.86 (p < 0.001) for all patients (Fig. 2b). At a cut-off of 172 mg/L (AUC of 86% (95% CI 78–93%), CRP had a sensitivity of 81% and a specificity of 76% for the detection of secondary bacterial infection. In patients with CRP < 172 mg/L, the negative predictive value was 90%, whereas CRP levels of > 172 mg/L had a positive predictive value of 62%.

## Discussion

The present study showed that PCT levels were normal in most patients with COVID-19 unless secondary bacterial infection was present. PCT levels < 0.55 ng/mL ruled out secondary bacterial infections with a negative predictive value of 94%. For CRP levels, the cut-off value was considerably elevated with 172 mg/L and predictive values slightly less robust. Inferring from this, PCT and CRP measurement may help identifying patients with secondary bacterial infections and allow a targeted use of antimicrobials thus promoting antibiotic stewardship.

Bacterial co-infections are uncommon in patients with mild COVID-19. However, secondary bacterial infections occur in an appreciable number of critically ill, hospitalized patients, since risk factors for nosocomial infections such as prolonged mechanical ventilation are prominent features of severe disease [17, 18]. Bacterial pneumonia, especially ventilator-associated pneumonia, is the most common secondary bacterial infection, but patients with severe COVID-19 are also susceptible to urinary tract and bloodstream infections [19].

On the other hand, many patients with COVID-19 have no secondary bacterial infection and don’t require antibiotic therapy. In a report from 552 hospitals in 30 Chinese provinces, 58% of patients were treated with antibiotics despite a low number of bacterial infections [2]. Antibiotics were administered to 80–100% of critically ill COVID-19 patients in Chinese ICUs [17, 20–22]. Based on past experience, clinicians often decide to take more antibiotic treatment for patients with severe illness. Empirical use of antibiotics is common but in the absence of bacterial co-infection, antibacterial therapy has no known benefit in patients with COVID-19 [10].

The presence of lower PCT levels has been shown to have a 94% negative predictive value for bacterial co-infection in intensive care unit patients with confirmed influenza A(H1N1)pdm09 [23]. Some biomarker studies have been conducted in hospitalized patients with COVID-19. Initial reports from China have shown that most patients with COVID-19 did not have elevated PCT (> 0.5 μg/L) [2, 17]. However, elevated levels were found more frequently in severe cases and in patients who died [2, 17, 24]. Our results showed, not surprisingly, that patients with secondary bacterial infections were more likely to have negative clinical outcomes than those with no evidence of bacterial infection. Even if CRP and PCT are also elevated in systemic response to COVID-19, the cut-off values in our study are much higher than described to discriminate between mild and severe disease [25]. In line with this observation, a recent review paper suggested that about 10% of deaths associated with viral disease are attributable to secondary bacterial infections [26].

In our study, a significant increase in both PCT and CRP levels was observed in patients with secondary bacterial infection. The receiver operated curve analysis of highest PCT and CRP yielded AUCs of 0.88 and 0.86, respectively. In patients with PCT < 0.55 ng/mL, the negative predictive value was 93%. With regard to antimicrobial stewardship, initiation of empirical antibacterial therapy in patients with low PCT can probably be avoided. CRP levels were also significantly higher in patients with bacterial infection but the cut-off value between both groups was with 172 mg/L considerably elevated (< 5 mg/L) and the predictive values slightly less robust. These results are in line with a recent investigation of Van Berkel et al. [9].

The available evidence together with our findings suggest that high proportion of COVID-19 patients received unnecessary antibiotic treatment [26, 27]. This increase in antibiotic administration can cause pressure on bacterial pathogens resulting antibiotic resistance [28, 29]. Hence, a potential consequence of the COVID-19 pandemic may be an accelerated propagation of antimicrobial resistance [30]. Clinicians, hospitals, microbiology labs, and public health organizations must be vigilant in monitoring the potential impact of increased antimicrobial consumption on emergence of resistance in individual patients and at institutional and regional levels.

Our study has several limitations, including the relatively small sample size, the monocentric setting, the retrospective design, the missing validation cohort, and the exclusion of patients with COVID-19 who were not hospitalized. In addition, the diagnosis of secondary bacterial infections was based not only on microbiology results but also on clinician’s decision about the relevance of the detected pathogen; e.g., bias and misclassifications cannot be excluded. It should also be noted that not all blood cultures, respiratory and urine samples were performed prior antibiotic treatment, which might influence the detection of bacterial infection.

In conclusion, PCT measurement on admission and during the course of the stay in patients with COVID-19 may help identifying secondary bacterial infections and allow for more targeted use of antimicrobial therapy. The use of CRP to detect secondary bacterial infection is also feasible but the cut-off value is considerably elevated. As the COVID-19 pandemic proceeds, prospective studies are needed to systematically collect clinical, microbiologic, and antimicrobial resistance data on superinfections. Results from carefully designed studies can be used to inform rational antimicrobial treatment and stewardship strategies, and to develop diagnostic criteria for secondary bacterial infections.

## Data Availability

Availability upon reasonable request.

## Abbreviations

AUC: Area under curve
BI: Betalactamase inhibitor
BC: Blood culture
BAL: Broncho alveolar lavage
CDI: *Clostridioides difficile* Infection
CoNS: Coagulase-negative staphylococci
CAP: Community-acquired pneumonia
COVID-19: Coronavirus disease 2019
CRP: C-reactive protein
GCP: Good clinical practice
ICU: Intensive care unit
LRTI: Lower respiratory tract infections
MHH: Medical School Hannover
MERS-CoV: Middle East respiratory syndrome coronavirus
POCT: Point-of-care testing
RT-PCR: Reverse transcription polymerase chain reaction
PCT: Procalcitonin
ROC: Receiver operating characteristic
SARS-CoV-2: Severe acute respiratory coronavirus 2
SARS-CoV-1: Severe acute respiratory coronavirus 1
SOP: Standard operation procedure
TS: Tracheal secret
TNF-α: Tumor necrosis factor-α
WBC: White blood count
WHO: World Health Organization
